# Clozapine rechallenge following neutropenia using granulocyte colony-stimulating factor: A Quebec case series

**DOI:** 10.1177/02698811211029737

**Published:** 2021-07-06

**Authors:** Laurent Béchard, Olivier Corbeil, Maude Plante, Marc-André Thivierge, Charles-Émile Lafrenière, Marc-André Roy, Marie-France Demers

**Affiliations:** 1 Faculté de pharmacie, Université Laval, Québec, Canada; 2 Institut universitaire en santé mentale de Québec, Centre intégré universitaire de santé et de services sociaux de la Capitale-Nationale, Quebec, Canada; 3 Centre intégré universitaire de santé et de services sociaux de l’Estrie – Centre hospitalier universitaire de Sherbrooke, Quebec, Canada; 4 Département de psychiatrie et neurosciences, Faculté de médecine, Université Laval, Canada; 5 Centre de recherche CERVO, Québec, Canada

**Keywords:** Clozapine, clozapine-induced agranulocytosis, clozapine-induced neutropenia, granulocyte colony-stimulating factor, rechallenge, schizophrenia

## Abstract

**Background:**

Clozapine has a unique efficacy profile among individuals suffering from treatment-resistant schizophrenia, but is associated with hematological side effects. The use of granulocyte colony-stimulating factors (G-CSF) to allow clozapine continuation or rechallenge has emerged as a promising option, but evidence is still scarce.

**Aim:**

To describe the largest case series so far published regarding this practice.

**Method:**

A national clozapine hematological monitoring database was consulted to identify all patients who had had neutrophil count <1.5 × 10^9^/L since 2004 in Quebec and was cross-referenced with hospital pharmacy software to identify patients who had received at least one dose of G-CSF, such as filgrastim, while being exposed to clozapine. All data were collected retrospectively, using patients’ medical files, from January to July 2019.

**Results:**

Using G-CSF, three out of eight patients could maintain clozapine despite neutropenia episodes that otherwise would have required treatment discontinuation. The only side effect reported was mild short-lived back pain, over a mean 3-year follow-up period. In all but one case, filgrastim was used on an “as-needed” basis at doses of 300 mcg administered subcutaneously.

**Conclusion:**

These results suggest that the “as-needed” use of G-CSF is well-tolerated and may allow clozapine rechallenge in some well-selected patients, adding to the paucity of data regarding long-term safety and efficacy of this strategy. More research may help to better define potential candidates and optimal regimen of such practice.

## Introduction

Clozapine is well known for its unique efficacy in patients with treatment-resistant schizophrenia (TRS), representing about 30% of this vulnerable population (Kane et al., 2019). Regardless, clozapine use in Canada is as low as 4–16%, mostly because of hematological concerns (Remington et al., 2016). Clozapine is associated, respectively, with 2–3% and 0.8% of cases of neutropenia and agranulocytosis (Alvir et al., 1993; de With et al., 2017). Although the underlying mechanism is not completely understood, it is believed that these side effects are immune-mediated, rather than toxic, and they do not appear to be dose-dependent (Alvir et al., 1993; Munro et al., 1999). They would be more frequent in females, in Africans/Asians, genetically predisposed individuals, within the first year of clozapine exposition, after a previous neutropenia/agranulocytosis episode or in the presence of other concomitant drugs also known to cause neutropenia (e.g. sodium valproate) (Alvir et al., 1993; Legge et al., 2018; Malik et al., 2018; Munro et al., 1999).

Upon monograph recommendations, clozapine requires mandatory hematological surveillance to prevent agranulocytosis and further complications. In Canada, an absolute neutrophil count (ANC) below 1.5 × 10^9^/L warrants for clozapine cessation and forbids its rechallenge (Mylan-Pharmaceuticals, 2020). In some patients, lowering hematological threshold is possible (e.g. benign ethnic neutropenia), but clozapine cessation may still be required upon reaching ANC value below the adjusted threshold (Sultan et al., 2017). In such situations, clozapine abrupt cessation is associated with an increased risk of sudden psychotic relapse, jeopardizing recovery (Atkinson et al., 2007; Miodownik et al., 2006). In some cases, seeking ways to reintroduce clozapine despite hematological caveats may provide the last chance of re-achieving recovery (Myles et al., 2017).

A brief review of the literature has highlighted two different off-label pharmacological strategies in Canada, used to re-expose a patient to clozapine. These strategies include the use of lithium to induce leukocytosis and the use of granulocyte colony-stimulating factor (G-CSF) to stimulate leukopoiesis (Boazak et al., 2018; Lally et al., 2017). These strategies may sometimes be used concomitantly, although it remains unknown whether such practice is superior to the use of both agents used separately. Furthermore, even though the use of G-CSF for chemotherapy-induced neutropenia is well documented, data regarding the use of G-CSF and lithium for clozapine-induced blood dyscrasias are scarce and are mainly based on case reports (Lally and Flanagan, 2016; Manu et al., 2018). For this latter purpose, G-CSF is either used “as-needed,” giving doses until neutrophil normalization only if an ANC result is below a prespecified threshold, or used as a “prophylactic strategy,” giving doses weekly on a regular basis independently of ANC values (Myles et al., 2017). In two recent systematic reviews, clozapine rechallenge using G-CSF showed a 75–76% success rate and an “as-needed” strategy seemed to be the most promising (Lally et al., 2017; Myles et al., 2017).

## Methods

In this paper, we describe the use of G-CSF to maintain clozapine despite neutropenia or agranulocytosis. A national clozapine hematological monitoring database was consulted to identify patients who had had neutropenia/agranulocytosis from January 2004 to January 2019 in every tertiary psychiatric hospital of Quebec. This information was cross-referenced through the hospitals’ pharmacy electronic databases to identify patients who had received at least one G-CSF dose while under clozapine treatment. Patients with an active cancer diagnosis at time of neutropenia were excluded. A group of Quebec pharmacists specialized in psychiatry was also contacted to ensure that every case in the province had been identified. A total of eight patients who had received G-CSF in order to allow either a clozapine rechallenge or its continuation despite neutropenia were identified in two psychiatric hospitals of Quebec, Canada. All data were collected from the patients’ medical files, from January to July 2019. This case series was approved by the local ethics and research committee. The *Clinical Global Impression severity* scale (CGI-S) was used to evaluate the disease severity during four critical turning points: before and after clozapine introduction, after a red result, and after clozapine rechallenge (Busner and Targum, 2007). The Naranjo algorithm was used to assess the likelihood of clozapine being responsible for the neutropenia/agranulocytosis for each episode (Naranjo et al., 1981).

## Results

### Case 1

Patient 1 has already been described in a previous case report (Demers et al., 2020). He is a 32-year-old Caucasian male diagnosed with schizophrenia at age 16 according to the Diagnostic and Statistical Manual of Mental Disorders version 5 (DSM-5). Six months following clozapine introduction, he developed neutropenia and clozapine was discontinued. During the following 2 years, he became severely psychotic and was hospitalized for a total of 161 non-consecutive days. Homicidal threats and aggressive behavior led to a clozapine rechallenge. A month later, he developed a second neutropenia; filgrastim was administered and ANC normalized. A week later, a second dose of filgrastim was required due to an ANC drop. Clozapine was maintained during those two episodes. The patient was not receiving any concomitant medication and there was no other contributory cause for these neutropenia episodes. The two doses of filgrastim were well tolerated and only a short-lived back pain was experienced. More than 8 years later, clozapine is still ongoing with positive outcomes.

### Case 2

A 65-year-old Caucasian female with DSM-5 schizophrenia diagnosed at the age of 22 started clozapine at the age of 47 for TRS, leading to a significant response. During the following 17 years, she experienced two mild neutropenia episodes that did not require clozapine discontinuation nor G-CSF use. A year later, she discontinued clozapine on her own because she felt physically ill. She was diagnosed with influenza and a possible bacterial pneumonia. Shortly after, she was admitted to the intensive care unit and received ceftriaxone while clozapine was being reinstated. She developed agranulocytosis 10 days after clozapine re-exposition. The drug was withdrawn, and she received a few doses of filgrastim to improve her ANC. Olanzapine was then initiated until a clozapine rechallenge was attempted 1 month later. After 12 days, she once again developed agranulocytosis; clozapine was withdrawn for good, unfortunately leading to a severe psychopathological deterioration.

### Case 3

A 45-year-old Caucasian male diagnosed with DSM-5 schizophrenia at the age of 28 had a first neutropenia after receiving clozapine for 14.5 years. Clozapine was withdrawn and a single dose of filgrastim was administered. The patient was also receiving haloperidol 10 mg daily, which was discontinued as well. ANC quickly normalized, and clozapine was reintroduced 12 days later. Clozapine treatment was still ongoing at follow-up.

### Case 4

A 53-year-old Caucasian male diagnosed with DSM-5 schizophrenia at the age of 22 had been hospitalized for the last 30 years due to TRS. Clozapine was only introduced at the age of 53 due to a low ANC at baseline (around 1.5 × 10^9^/L). Noteworthy, he had received a single dose of clozapine by mistake in his 20s, which led to an ANC drop. However, at that time, the patient was also taking sodium valproate, chlorpromazine and olanzapine. Three months after clozapine initiation, while also receiving quetiapine, a first neutropenia occurred. Clozapine was discontinued but reintroduced 8 months later with a lowered ANC surveillance threshold given his low baseline values (from 1.5 to 1.0 and eventually to 0.8 × 10^9^/L). Despite this adjustment, the patient had nine other neutropenia episodes during which clozapine could nonetheless be maintained using a total of 13 “as-needed” doses of filgrastim over 3 months. Following a 10^th^ neutropenia, clozapine was discontinued because of severe agranulocytosis.

### Case 5

A 21-year-old Caucasian female diagnosed with DSM-5 schizophrenia at the age of 20 started clozapine early on following her diagnosis due to a significant substance use disorder history and TRS. She developed neutropenia 4 months later while also taking haloperidol, which was discontinued. Clozapine was maintained, even after a second neutropenia 6 days later, given that in both instances, blood tests repeated in the afternoon showed normal ANC values. After a third episode a few days later, confirmed by an afternoon blood test, a single dose of filgrastim was administered and ANC quickly normalized. Clozapine was maintained and is still ongoing at follow-up.

### Case 6

A 60-year-old Caucasian male diagnosed with DSM-5 schizophrenia and potomania at the age of 19 was receiving clozapine for 10 years when a first neutropenia occurred. Clozapine was discontinued, leading to numerous psychiatric hospitalizations during the following 2 years. As a result, clozapine was rechallenged with a reduced ANC surveillance threshold (1.0 × 10^9^/L). Two months later, the patient experienced two neutropenia episodes in a row. At that time, the patient was also taking quetiapine, oxazepam, and citalopram. Two doses of filgrastim were given during the last episode, a few days apart, which only led to transitory ANC normalization. Clozapine was withdrawn shortly after as the medical team feared the undocumented innocuity of filgrastim in this off-label use.

### Case 7

A 53-year-old Caucasian male diagnosed with DSM-5 schizoaffective disorder started clozapine at the age of 43 despite low baseline ANC values. While also receiving sodium valproate, neutropenia occurred 8 years later, leading to clozapine discontinuation. The patient eventually developed thrombocytopenia secondary to sodium valproate, which was switched for lithium. A year after, clozapine was reintroduced with a lowered ANC surveillance threshold. Only 3 months later, ANC dropped below the adjusted threshold and a hematologist suggested prophylactic use of filgrastim, initially twice weekly, and then thrice weekly for a year to allow clozapine to be maintained. G-CSF was eventually stopped due to recurrent leucocytosis, resulting in agranulocytosis 20 days later. Clozapine was stopped and G-CSF was reinstated, with ANC normalization; unfortunately, the patient died from choking on an apple 14 days later and it was concluded that the cause of death was unrelated to agranulocytosis, clozapine or G-CSF.

### Case 8

This 54-year-old Caucasian woman was hospitalized for 17 years because of refractory epilepsy and DSM-5 schizophrenia that was diagnosed at the age of 33. She was known to have low baseline ANC values and she had also previously experienced idiosyncratic leucopenia, thrombocytopenia, as well as pancytopenia due to carbamazepine, sodium valproate and lamotrigine. When clozapine was introduced, patient 8 was receiving periciazine and lithium; the latter was withdrawn 2 years later due to intoxication, 6 days after which neutropenia occurred. ANC quickly normalized and clozapine could be maintained. A second neutropenia developed 9 months later, for which the patient received 3 consecutive filgrastim doses to allow clozapine treatment continuation. The same filgrastim strategy was subsequently required on 2 other occasions during the following 6 months to maintain clozapine, until it had to be temporarily held for a short period due to hepatitis. A month later, clozapine was gradually reintroduced with a reduced ANC threshold of 1.2 x 10^9^/L. The patient’s mental state was stable for the following 3.7 years until neutropenia reoccurred. This episode required one dose of filgrastim and a month later, clozapine had to be discontinued for good due to another neutropenia.

## Discussion

To our knowledge, this is the largest case series of clozapine rechallenge with the use of G-CSF. Overall, 38% (3/8) patients remained on clozapine treatment after a mean follow-up period of 3 years using only 1-2 G-CSF doses (see Table 1). There were no deaths nor any infectious complications related to clozapine.

**Table 1.** Patients characteristics.

	Demographics^a,b^	Neutropenia episode	Time to neutropenia episode^c^	ANC Nadir (×10^9^/L)	G-CSF protocol	Time before rechallenge^d^	Follow-up^e^	Continued clozapine use at follow up
Patient 1	21, Male	First	6 months	0.84	No filgrastim used	2 years	8.71 years	Yes
		Second	1 month	0.58	Filgrastim 300 µg daily if ANC < 1.0 (2 doses were administered)	Continued		
Patient 2	58, Female	First	12.2 years	1.22	No filgrastim used	Continued	0.85 year	No
		Second	4.16 years	1.22	No filgrastim used	Continued		
		Third	10 days	0.38	Filgrastim 300 µg daily until ANC > 0.5 (6 doses were used)	34 days		
		Fourth	12 days	0.32	Filgrastim 300 µg daily until ANC > 0.5 (2 doses were used)	Stopped		
Patient 3	45, Male	First	14.5 years	0.84	Filgrastim 300 µg × 1 dose	12 days	3.07 years	Yes
Patient 4	53, Male	First	3 months	0.65	No filgrastim used	8 months	3.08 years	No
		Second	10 months	0.81	No filgrastim used	Continued		
		Third	1 month	0.54	Filgrastim 300 µg daily if ANC < 0.8 (2 doses were used)	Continued		
		Fourth	7 days	0.74	*Idem* (1 dose was used)	Continued		
		Fifth	5 days	0.69	*Idem* (1 dose was used)	Continued		
		Sixth	17 days	0.62	*Idem* (1 dose was used)	Continued		
		Seventh	8 days	0.55	*Idem* (1 dose was used)	Continued		
		Eighth	6 days	0.75	*Idem* (1 dose was used)	Continued		
		Ninth	6 days	0.79	*Idem* (1 dose was used)	Continued		
		Tenth	24 days	0.11	Filgrastim 300 µg daily if ANC < 1.0 (5 doses were used)	Stopped		
Patient 5	21, Female	First	4 months	1.33	No filgrastim used	Continued	1.64 years	Yes
		Second	6 days	1.36	No filgrastim used	Continued		
		Third	10 days	1.07	Filgrastim 300 µg × 1 dose	Continued		
Patient 6	60, Male	First	10 years	0.85	No filgrastim used	2 years	3.08 years	No
		Second	2 months	1.3	No filgrastim used	Continued		
		Third	6 days	1.1	Filgrastim 300 µg × 2 doses	Stopped		
Patient 7	51, Male	First	7.8 years	1.26	No filgrastim used	1 year	14 days	No
		Second	3.5 months	NS	Filgrastim 300 µg 3 ×/week for a year	Continued		
		Third	1.4 years	0.3	Filgrastim 300 µg daily until ANC normalization	Stopped		
Patient 8	42, Female	First	1.8 years	1.1	No filgrastim used	Continued	3.5 years	No
		Second	9 months	1.0	Filgrastim 300 µg 3 ×/week until ANC > 1.5 (3 doses were used)	Continued		
		Third	25 days	1.3	Filgrastim 300 µg 3 ×/week until ANC > 1.5 (3 doses were used)	Continued		
		Fourth	4.5 months	1.3	Filgrastim 300 µg 3 ×/week until ANC > 1.5 (3 doses were used)	Continued		
		Fifth	3.7 years	0.8	Filgrastim 300 µg 3 ×/week until ANC > 1.2 (1 dose was used)	Continued		
		Sixth	26 days	1.1	No filgrastim used	Stopped		

Table 1 is meant to be read from left to right. Each line represents a neutropenia episode.

ANC: absolute neutrophil count; G-CSF: granulocyte colony-stimulating factor.

a The age in the demographics is the age at the first neutropenia episode.

b All patients were diagnosed with schizophrenia or schizophrenia spectrum disorder and all patients were Caucasian.

c Time to neutropenia episode: time from clozapine initiation or from last neutropenia episode (if clozapine was pursued despite neutropenia) to the corresponding neutropenia episode.

d Time before rechallenge: If clozapine was stopped following the neutropenia episode, time from the cessation to rechallenge.

e Time of follow-up is the time from the last clozapine rechallenge or withdrawal to July 2019 or death.

All patients were Caucasian and median age at first neutropenia was 48 years (see Table 1). We observed five late-onset neutropenia (i.e. > 1 years), with a median time of 4.8 years before the initial neutropenia. This is rather surprising considering the rare incidence of neutropenia after the first year of clozapine treatment. Perhaps this could be explained by the fact that clinicians are more likely to rechallenge clozapine in patients experiencing substantial clinical improvement over a long time. On the contrary, clinicians might hesitate to rechallenge patients whose initial clozapine trial was stopped prematurely due to neutropenia, without a well-established long-term response.

A mean number of 4 (ranging from one to ten events) neutropenia events were observed for each patient. Almost every patient (7/8) had one or more subsequent ANC drop following the initial neutropenia. For these patients, as previously observed in other studies, subsequent neutropenia episodes developed quicker following clozapine rechallenge, with lower mean ANC at nadir for the third and subsequent neutropenia episode in comparison to the first (Dunk et al., 2006).

Lithium use to induce leucocytosis seems to be a common practice in Quebec as it was used at some point in 7/8 cases while it was only used in 5/30 cases in a previous systematic review (Myles et al., 2017).

As for G-CSF use, 7/8 patients had an “as-needed” strategy with a pre-established ANC threshold varying from 0.5 × 10^9^/L to 1.5 × 10^9^/L. Intervals between doses and number of doses received by patients varied extensively (see Table 1). Patient 7 was the only one receiving G-CSF with a prophylactic strategy. He received regular doses of G-CSF for a year with an adequate hematologic response during that time. G-CSF prescription patterns reported herein thus differ from previously published cases where G-CSF was most commonly given using a prophylactic protocol (Myles et al., 2017). However, in contrast with a previous systematic review that reported a 100% success rate for clozapine rechallenge associated with the “as-needed” use of G-CSF, that strategy was successful in only three out of seven cases (Myles et al., 2017). The “as-needed” strategies used in this case series were identical in terms of ANC threshold to the identified cases in Myles and colleagues’ review but, differed slightly in terms of G-CSF agents used (Myles et al., 2017). Given that a younger age is associated with an increased rechallenge success rate, this discrepancy could in part be accounted by the fact that patients described here were on average more than 10 years older than previously published case reports (Meyer et al., 2015; Myles et al., 2017). Consequently, considering the small number of published cases and the potential for publication bias, uncertainty remains as to which G-CSF strategy should be favored.

In all cases, aside from leucocytosis, G-CSF was well tolerated, with only 1 patient reporting short-lived back pain.

As for the timeline of use, G-CSF was given at the first occurrence of neutropenia solely for patient 3 resulting in a rapid ANC normalization and a quick rechallenge. In contrast, 4/8 patients did not use G-CSF at the first neutropenia episode and stopped clozapine for an average time of 16 months before a rechallenge was attempted, which is longer than the 9 months median time reported by Myles and colleagues’ (Myles et al., 2017). This leads to wonder if an earlier use of G-CSF could provide better results. Three patients continued clozapine despite neutropenia without initially resorting to G-CSF. The average follow-up of 4.5 years after successful clozapine rechallenge indicates that our results are durable.

Although clozapine rechallenge following agranulocytosis seems to pose more risks and may have lower success rates according to a recent systematic review, many questions remain unanswered regarding predictive factors for clozapine rechallenge (Manu et al., 2018). Likewise, a previous study reported a 22-fold increased risk of agranulocytosis following clozapine rechallenge independently of the presence of other possible contributory causes for the first neutropenia episode (Dunk et al., 2006). However, this finding was based on only a few number of patients and has not been reproduced. Hence, assessing the likelihood that clozapine is in fact the cause of a neutropenia episode is still clinically relevant. For those reasons, clozapine’s imputability in all neutropenia episodes was carefully assessed using the Naranjo algorithm, a validated and widely used pharmacovigilance tool (Naranjo et al., 1981). Although, clozapine was considered to be the most probable cause in most cases, it was only identified as a definite cause with no other explanation for patients one and five. Interestingly, they were cases of successful clozapine rechallenge. Other possible causes included low ANC at baseline, infections and the use of concomitant drugs known to cause neutropenia, namely here, benzodiazepines, proton pump inhibitors, antidepressants, antipsychotics, ceftriaxone, gabapentin, and sodium valproate (Lally and Flanagan, 2016; Porter and Mohamed, 2006).

One thing is certain, clozapine cessation has immense consequences on psychopathology and recovery (Atkinson et al., 2007; Miodownik et al., 2006). Indeed, the mean CGI-S score before clozapine was 5.5, despite ongoing antipsychotic treatment. Clozapine initiation significantly improved psychopathology, as the mean CGI-S decreased to 3.4 and all patients had scores ⩽ 4 while receiving clozapine. As previously published, clozapine cessation frequently leads to psychotic relapse, and symptom severity may sometimes exceed that of the symptoms experienced before using clozapine, as was the case for patients one and two (Meltzer et al., 1996). Further, the mean CGI-S score for patients who had to stop clozapine was 6.2. Fortunately, patients who were successfully rechallenged with clozapine had a strong clinical improvement and benefited from a response that was quite similar to that of the first clozapine trial.

In conclusion, to our knowledge, there are no death reports following clozapine rechallenge using G-CSF. Furthermore, G-CSF is generally well-tolerated and very few side effects have been reported so far from its use in psychiatry (Myles et al., 2017). Also, patient 7 as well as other documented cases have used both clozapine and G-CSF for prolonged periods of time without any major side effects, suggesting long-term safety (Hägg et al., 2003; Mathewson and Lindenmayer, 2007). Keeping in mind the impact of clozapine withdrawal on patients’ recovery and the increased likelihood of recurrent neutropenia being more severe during rechallenges, patients might benefit from earlier use of G-CSF. However, more evidence is needed to provide clear guidelines regarding both clozapine rechallenge and the use of G-CSF in that context, including the prescribing strategy to be preferred. Furthermore, while it is more common to attempt clozapine rechallenges only for patients who might have had other contributory causes at time of first neutropenia, patients for whom clozapine was the sole cause might still benefit from a rechallenge, as it was done for some of our cases. Although more research regarding this latter is evidently needed, perhaps clozapine rechallenge using G-CSF should become a more common practice for some well-selected patients.
